# Clinical Manifestations, Diagnosis, Treatment and Prognosis of Uveitis Induced by Anticancer Drugs: A Review of Literature

**DOI:** 10.3390/brainsci12091168

**Published:** 2022-08-31

**Authors:** Dongchen Li, Li Yang, Feng Bai, Shun Zeng, Xiaoli Liu

**Affiliations:** 1 Ophthalmologic Center of the Second Hospital, Jilin University, Ziqiang Street 218, Changchun 130000, China; 2 Clinical College, Jilin University, Xinmin Street 70, Changchun 130000, China

**Keywords:** uveitis induced by anticancer drugs, immunotherapy, specific targeted therapy

## Abstract

There are increasing reports that anticancer drugs, especially immunotherapy and specific targeted therapy, can cause uveitis, but it is not fully understood whether the clinical features of this drug-induced uveitis differ from those of other types of uveitis and whether there are differences between these drugs. We retrospectively reviewed the published cases and case series in PubMed, Embase, Web of Science, and Cochrane from January 2011 to October 2020. We analysed the data, including patients’ basic information, medications used, duration of use, time to onset, clinical manifestations, diagnosis, treatment, and prognosis of uveitis. We focused on the differences in uveitis caused by immunotherapy and specific targeted therapy. Altogether 93 cases (43 men, 48 women, and 2 cases whose gender was not mentioned) reported in 55 articles were included in this study. The average age was 59.6 ± 13.5 years. Eighty percent of the patients had bilateral involvement. Sixty cases were caused by immunotherapy (64.5%), and twenty-six were caused by specific targeted therapy (27.9%). No significant difference was found in the mean time from treatment to onset between the two groups. Anticancer drug-induced uveitis can involve all parts of the uvea from anterior to posterior, manifested as anterior chamber flare, anterior chamber cells, papillitis, macular oedema, subretinal fluid, and choroidal effusion. Anterior uveitis (24 cases, 40.0%) was more common in immunotherapy, and intermediate uveitis (8 cases, 30.8%) was more common in specific targeted therapy. The mean LogMAR visual acuity in specific targeted therapy at presentation was lower than in immunotherapy, but it was not statistically significant. Corticosteroid therapy can effectively control uveitis induced by anticancer drugs. However, the survival prognosis was poor. Among the 19 patients with reported cancer prognosis, seven (36.8%) had no cancer progression, eight (42.1%) had further metastases, and four (21.0%) died of cancer. In conclusion, uveitis caused by anticancer drugs involves both eyes and manifests as various types of uveitis. Patients with specific targeted therapy are more likely to have intermediate uveitis and low vision, and immunotherapy patients are more likely to have anterior uveitis. Corticosteroids are effective against uveitis caused by anticancer drugs.

## 1. Introduction

At present, immunotherapy and targeted therapy have been widely used to treat cancer and greatly extended the life of cancer patients [1,2]. Specific targeted therapies exert antitumour effects by inhibiting abnormally expressed signalling pathways that cause tumorigenesis. Cytotoxic T lymphocyte-associated antigen-4 (CTLA-4) and programmed cell death protein-1 (PD-1) activate two negative regulatory molecules on the surface of T cells and inhibit the activation of T cells. Antibodies against CTLA-4 and PD-1 can block these two molecules that are highly expressed on the surface of cancer cells, promote T cell activation, and then kill cancer cells. It should not be ignored that a small number of patients who use these anticancer medicines develop immune-related diseases including uveitis [3]. For example, unique ocular toxic events have been observed in patients receiving targeted therapy with MEK inhibitors [4]. More than 60% of patients treated with immunotherapeutic agents have side effects, which are called immune-related adverse events (irAEs) [5,6]. Among them, skin and gastrointestinal tract side effects are the most common. [5] Ocular side effects including uveitis are generally rare but require attention [5]. Bacillus Calmette-Guérin (BCG), which is used to treat bladder tumours, can cause uveitis, endophthalmitis, and a rare form of choroiditis because of an allergic reaction or direct bacterial invasion of the choroid [7]. Prompt recognition of uveitis is critical to the care of patients receiving anticancer drugs including immune checkpoint inhibitors [8]. Until now, it has not been fully understood whether the clinical features of this drug-induced uveitis differ from those of other types of uveitis and whether there are differences between these anticancer drugs. In this paper, we reviewed case reports on anticancer-drugs-induced uveitis from 2011 to 2020 and analysed patient information, clinical manifestations, diagnosis, treatment, and clinical and visual outcomes.

## 2. Patients and Methods

**Figure 1:**
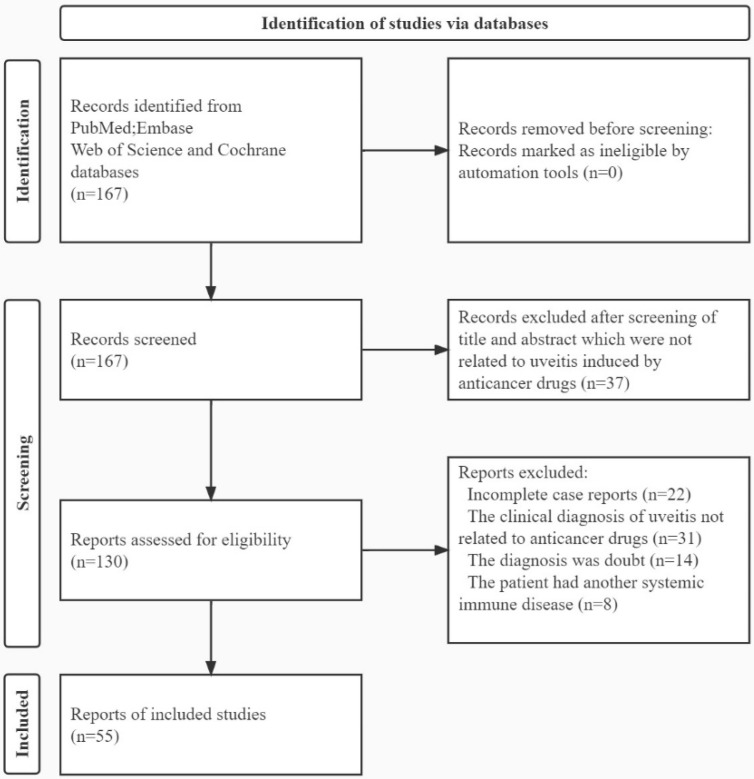
The flowchart for identification of articles in databases.

The search queries “Neoplasms/drug therapy” [Mesh] and “Uveitis” were used to search for articles in PubMed, Embase, Web of Science, and Cochrane. The literature from January 2011 to October 2020 was included. The studies were included only if there was a clinical diagnosis of uveitis related to anticancer drugs. If the diagnosis was in doubt, no diagnosis was made, there was no clear relationship between uveitis and anticancer drugs, or the patient had another systemic immune disease, the paper was excluded (Figure 1). We collected all the basic information, including age and sex of the patient; type of cancer; time to occurrence of uveitis; clinical information of uveitis, including the manifestations under the slit lamp, fundus examination and auxiliary examination; the course of treatment, prognosis, and whether the use of anticancer agents was halted. The anatomical classification was conducted according to the standardisation of uveitis nomenclature (SUN) as anterior uveitis, intermediate uveitis, posterior uveitis, or panuveitis [9]. The patient’s visual acuity was converted to a logarithm of the minimum angle of resolution (LogMAR). The hand motion record was 3.0, the light perception record was 4.0, and the no light perception record was 5.0. To study the clinical features of uveitis caused by different anticancer drugs, we divided all cases into three groups, immunotherapy, specific targeted therapy, and other anticancer drugs. The clinical characteristics of uveitis in patients were described by calculating the frequency of each feature in these cases. Chi-square and Fisher exact tests (Software SPSS Statistics 28.0, International Business Machines Corporation, New York, NY, USA) were used to compare the differences in uveitis between the immunotherapy and specific targeted therapy groups. Independent sample t-tests were used to compare the visual acuity at presentation and the time from treatment to onset between the two groups. *p* values of less than 0.05 were considered statistically significant. This study met the requirements of the Declaration of Helsinki and was approved by the Clinical Ethics Committee.

## 3. Results

### 3.1. Epidemiology

From 2011 to 2020, 93 cases of uveitis were reported in 55 articles [1,2,3,4,5,6,7,10,11,12,13,14,15,16,17,18,19,20,21,22,23,24,25,26,27,28,29,30,31,32,33,34,35,36,37,38,39,40,41,42,43,44,45,46,47,48,49,50,51,52,53,54,55,56,57]. Among them, there were 43 men (46.9%) and 48 women (50.6%). The average age was 59.2 ± 12.8 years, with a range of 7–92 years. The average age of the men was 59.1 ± 11.1 years and of the women was 59.3 ± 14.5 years. There were two 60-year-old patients whose gender was not mentioned.

### 3.2. Aetiology

Uveitis was caused by immunotherapy (60 cases, 64.5%) and specific targeted therapy (26 cases, 27.9%). Immunotherapy-induced uveitis involved anti-PD-1 agents (pembrolizumab (23 cases, 24.7%) and nivolumab (13 cases, 14.0%)) and T-lymphocyte antigen-4 (ipilimumab (18 cases, 19.4%) plus ipilimumab and nivolumab (6 cases, 6.5%)). Specific targeted therapies included BRAF inhibitor vemurafenib (12 cases, 13.0%); MEK inhibitors (XL518 (3 cases, 3.2%) and encorafenib and binimetinib (1 case, 1.1%)); BRAF inhibitor combined with MEK inhibitor (trametinib and dabrafenib (6 cases, 6.5%)); inhibitor of epidermal growth factor receptor tyrosine kinase (erlotinib (3 cases, 3.2%)); and C-kit inhibitor (nilotinib (1 case, 1.1%)). In addition, the BCG vaccine used for bladder cancer (6 cases, 6.5%) might also have caused uveal inflammation. Uveitis had also been reported with anastrozole prescribed for breast cancer (1 case, 1.1%). The indications for anticancer drugs to treat cancer are shown in Table 1.

### 3.3. Clinical Character

Eighty-four per cent of patients had bilateral involvement. Both eyes were simultaneously affected in 80.0% of patients in the immunotherapy group and in 84.6% of patients in the specific targeted therapy group. The time span from treatment to onset was wide-ranging, with the mean time of 13.8 ± 19.4 weeks for the immunotherapy group, 22.8 ± 22.5 weeks for nivolumab, and 15.3 ± 22.8 weeks for pembrolizumab. The time span from treatment to onset for the specific targeted therapy group was longer, with the mean time of 21.6 ± 22.7 weeks and 39.9 ± 38.6 weeks for vemurafenib. The average with BCG was 11.17 ± 7.67 weeks. Patients developed uveitis more than two years after using anastrozole.

Visual acuity was recorded in 52 patients (55.9%), and the mean LogMAR acuity was 0.48 ± 0.94 at presentation. The mean LogMAR visual acuity of the immunotherapy and specific targeted therapy groups was 0.34 ± 0.47 and 0.74 ± 1.38, respectively. Of the 18 patients with recorded IOP, 3 had elevated IOP (>21 mmHg). One ipilimumab patient had 35 mmHg in the right eye and 43 mmHg in the left eye [5], and one nivolumab patient had elevated IOP [41]. One patient developed elevated intraocular pressure secondary to the pupil block due to vemurafenib [48]. Three patients had an IOP of less than or equal to 10 mmHg: two patients receiving pembrolizumab had an IOP of 6 mmHg and 8 mmHg [15,38], respectively, and one patient receiving BCG had an IOP of 10 mmHg [46]. The average intraocular pressure was 16.32 ± 11.97 mmHg.

Decreased vision was the most common initial symptom. In the immunotherapy group, nearly 28.3% of the patients’ first visit was due to decreased vision, while in the specific targeted therapy group, it was 53.9%. Statistical analysis showed that it was significantly higher in the specific targeted therapy group than in the immunotherapy group. Other ocular manifestations, such as redness and eye pain (19.2%) and floaters (7.7%), were found in both two groups. A total of 16.7% of the cases were accompanied by systemic manifestations in the immunotherapy group, of which VKH-like systemic manifestations were the most common (40.0%), while 11.5% of patients had systemic manifestations in the specific targeted therapy group. Both groups could involve all parts of the uvea from anterior to posterior. Intermediate uveitis (8 cases, 30.8%) was more common in specific targeted therapy (*p* < 0.05). In terms of fundus changes, papillitis (18.3% and 7.7%), retinal detachment (8.3% and 23.1%), and macular oedema (15.0% and 15.4%) occurred in the immunotherapy and specific targeted therapy groups, respectively, and no significant difference was found. In the immunotherapy group, the most common causes of uveitis were pembrolizumab (23, 24.7%) and ipilimumab (18, 19.4%). In the specific targeted therapy group, the most common cause of uveitis was vemurafenib, which was dominated by ocular symptoms and signs of the anterior segment. Changes in the fundus were mainly macular oedema (Table 2, Table 3 and Table 4).

### 3.4. Treatment

Of the 91 patients with treatment records, in 36 cases (39.6%) it was chosen to discontinue medication. Anti-inflammatory therapy was the first option considered, and 79 patients were treated with steroids, of which 44 patients (55.7%) were treated with topical therapy, 16 patients (20.3%) were treated with a combination of topical and systemic therapy, and 18 patients (22.8%) were treated with systemic therapy. Prednisolone was the most commonly used drug in systemic therapy (15 cases, 18.9%). Prednisone acetate was the most commonly used drug in topical therapy (12 cases, 15.2%). Other corticosteroids included dexamethasone (11 cases, 13.9%), methylprednisolone (11 cases, 13.9%), triamcinolone acetonide (7 cases, 8.9%), and betamethasone (3 cases, 3.8%). Another 12 patients selected other anti-inflammatory agents: seven patients received difluprednate, a difluorinated derivative of prednisolone that has strong anti-inflammatory activity; four patients received topical virostatic drug such as acyclovir for one month; non-steroidal anti-inflammatory drugs such as bromfenac sodium were also used in anti-inflammatory treatment.

In addition to receiving anti-inflammatory treatment, patients with different symptoms received different treatments. If posterior synechiae were present (4 cases), atropine (2 cases), scopolamine (1 case), or cyclopentolate (1 case) might be used. At the same time, subconjunctival injection of sympathetic agents (2 cases) could rupture the adhesion. Patients with an elevated IOP may be treated with IOP-lowering agents [5], and one patient with an IOP over 50 mmHg received intravenous mannitol [36]. An intravitreal injection of ranibizumab could be used to treat choroidal neovascularisation secondary to multifocal choroiditis [13]. Some patients with bladder cancer who received BCG developed an ocular inflammation with cystic macular oedema (CME) and submacular fluid [31]. After other possible causes were ruled out, it was decided to start 2 months of anti-tuberculous therapy (ATT) with isoniazid (300 mg/day), rifampin (600 mg/day), and ethambutol (1200 mg/day), and inflammation rapidly decreased to 0+, and macular degeneration subsided at 3 weeks [31]. However, it is worth noting that if the pupillary block caused by posterior synechiae was too severe, surgical peripheral iridectomy was required.

### 3.5. Prognosis

The majority of patients had a good prognosis, and uveitis was cured in an average of 3.6 ± 4.4 months after treatment. Only two patients had recurrent uveitis without discontinuation of the medication. In contrast with the prognosis for uveitis, the patient’s cancer prognosis was poor. Of the 19 cases reported, only seven cases (36.8%) had no cancer progression, eight cases (42.1%) had further metastasis, and four cases (21.0%) died of cancer shortly thereafter. Of the 19 patients with documented survival outcomes, 12 discontinued anticancer drugs because of uveitis, and their survival outcomes were worse, with 67% having further cancer progression or death. A patient with choroidal melanoma of the right eye for more than 3 years died of progressive metastatic omental disease 6 weeks after the onset of uveitis. A patient with metastatic cutaneous melanoma for over 8 months died of systemic complications of metastatic melanoma 6 months after uveitis. A patient with stage IV squamous cell cancer of the left lung for 15 months with liver metastasis died 7 months after the onset of uveitis. A patient with a four-year malignant melanoma course died four months after uveitis.

## 4. Discussion

The management of uveitis in patients treated with anticancer drugs is complex. One major challenge was to determine whether uveitis was caused by the oncologic treatment or by other possible causes. Uveitis caused by anticancer drugs could be inferred from chronological order. For example, ocular and systemic symptoms occurred simultaneously after 5 weeks of erlotinib treatment [30]. Bilateral uveitis and papillitis started after 2 months of pembrolizumab treatment and improved significantly after discontinuation and initiation of topical steroid therapy. When pembrolizumab was restarted, bilateral uveitis recurred [11]. Other causes of uveitis could be ruled out by extensive examination. Multimodal imaging using optical coherence tomography (OCT), fundus fluorescein angiography (FA), and indocyanine green angiography (ICGA) could provide detailed ocular features of uveitis. Blood tests were also used to screen for the cause of uveitis.

The clinical features of uveitis caused by anticancer drugs were related to the drugs used, regardless of the age or sex of the patients. More than 95% of patients presented with simultaneous uveitis in both eyes. Immunotherapy and specific targeted therapy can cause panuveitis, manifested as anterior chamber flare, anterior chamber cells, papillitis, macular oedema, and subretinal fluid. In both groups, choroidal effusion had been reported. However, patients in the targeted drug treatment group were more likely to experience decreased vision, redness, and pain. This was associated with higher rates of posterior synechiae and serous retinal detachment in the targeted drug group. It was suggested that uveitis induced by specific targeted therapy might be more serious. The time span from the initiation of anticancer drugs to the appearance of uveitis was slightly longer in the specific targeted therapy group, especially for vemurafenib, reaching 32.78 ± 32.14 weeks. Ipilimumab was more likely to cause uveitis, with uveitis occurring after an average of 4.31 ± 3.15 weeks. The immunotherapy group was more prone to systemic changes such as VKH-like vitiligo [17]. Every patient (no matter if the patient was on immune or specific targeted therapy) must be examined completely, both with anterior and posterior segment examinations.

The treatment of uveitis induced by anticancer drugs needs to be determined on a case-by-case basis. The most commonly used drugs for treatment were steroids. Severe episodes of uveitis may require withdrawal and systemic corticosteroid treatment, such as short-term intravenous methylprednisolone, followed by oral prednisolone. However, most patients had mild uveitis, and systemic corticosteroids were not recommended because of tumour therapy, so topical corticosteroids such as topical prednisone 1% acetate drops (eight times per day) should be used. If there was no active disease and vision was normal, routine monitoring of uveitis was recommended, and corticosteroid treatment might be appropriately postponed. Nonsteroidal anti-inflammatory drugs (bromfenac sodium) can be used to control inflammation [2]. Others include symptomatic treatment. Mydriatic drugs were used to prevent and treat posterior synechiae. For patients with elevated intraocular pressure, intraocular pressure-lowering drugs should be concomitantly used. When choroidal neovascularisation was present, intravitreal injection of anti-VEGF agents such as ranibizumab should be performed. Some patients with bladder cancer who receive BCG injections may be treated with anti-tuberculous treatment after other possible causes have been ruled out [31].

In conclusion, anticancer drugs, including both immunotherapy and specific targeted therapy, could induce uveitis. There is no statistically significant difference in the time interval from the start of medication to the appearance of uveitis between the two groups. Uveitis caused in either of the two groups might be accompanied by systemic changes. Both therapies can cause various fundus changes, including serous retinal detachment. After symptomatic treatment using corticosteroids, most patients with uveitis have a good prognosis. Given that anticancer drugs can induce uveitis, it is recommended that patients taking these drugs should be routinely screened by a uveitis specialist.

## Figures and Tables

**Table 1:** Indications of tumour drugs mentioned in the literature.

Classification	Medicine	Cancer
Immunotherapy	Ipilimumab	Metastatic melanoma
	Nivolumab	Metastatic melanoma
		Non-small cell lung cancer
		Hodgkin’s lymphoma
		Bronchial adenocarcinoma
		Renal cell carcinoma
	Pembrolizumab	Metastatic melanoma
		Non-small cell lung cancer
	Ipilimumab and Nivolumab	Metastatic melanoma
		Endometrial cancer
Specific targeted therapy	Erlotinib	Non-small cell lung cancer
		Squamous cell cancer
	Trametinib and Dabrafenib	Metastatic melanoma
	Vemurafenib	Metastatic melanoma
	Nilotinib	Myeloid leukaemia
	MEK Inhibitor XL518	Metastatic melanoma
		Myeloid leukaemia
	Encorafenb and Binimetinib	Metastatic melanoma
Others	Bacillus Calmette-Guérin	Bladder carcinoma
	Anastrozole	Breast carcinoma

MEK: mitogen-activated protein kinase kinase.

**Table 2:** Clinical manifestations of uveitis caused by immunotherapy.

Clinical Manifestations		Ipilimumab	Nivolumab	Pembrolizumab	Ipi-Nivo	Total
Total, n		18	13	23	6	60
Sex	Male, n (%)	7 (38.89%)	4 (30.77%)	13 (56.53%)	4 (66.67%)	28 (46.67%)
	Female, n (%)	11 (61.11%)	9 (69.23%)	8 (34.78%)	2 (33.33%)	30 (50.00%)
Mean Age, years ± SD		56.17 ± 11.89	60.15 ± 12.68	61.09 ± 15.58	57.17 ± 7.69	59.02 ± 13.46
Bilateral involvement, n (%)		15 (83.33%)	11 (84.62%)	16 (69.57%)	6 (100%)	48 (80.00%)
Simultaneous onset of both eyes, n (%)		14 (77.78%)	10 (76.92%)	16 (69.57%)	6 (100%)	46 (76.67%)
Mean time from the initiation of treatment to onset of uveitis, weeks ± SD		4.31 ± 3.15	22.83 ± 22.52	15.25 ± 22.83	11.67 ± 7.99	13.82 ± 19.42
First symptoms	Vision loss, n (%)	5 (27.78%)	2 (15.38%)	10 (43.48%)		17 (28.33%)
	Red and pain, n (%)	3 (16.67%)	3 (23.08%)	2 (8.70%)		8 (13.33%)
	Floaters, n (%)	2 (11.11%)	1 (7.69%)	2 (8.70%)		5 (8.33%)
	Visual field defect, n (%)	1 (5.56%)				1 (1.67%)
systemic condition	VKH-like systemic manifestations, n (%)	1 (5.56%)	2 (15.38%)	1 (4.35%)		4 (6.67%)
	Hypophysitis, n (%)	3 (16.67%)				3 (5.00%)
	colitis, n (%)	1 (5.56%)		1 (4.35%)		2 (3.33%)
	Autoimmune hepatitis, n (%)	1 (5.56%)				1 (1.67%)
	pain, n (%)					
Anterior segment	posterior synechiae, n (%)	2 (11.11%)	3 (23.08%)	1 (4.35%)	1 (16.67%)	7 (11.67%)
	keratic precipitates, n (%)	2 (11.11%)	2 (15.38%)	1 (4.35%)		5 (8.33%)
	fibrin formation, n (%)	1 (5.56%)	1 (7.69%)			2 (3.33%)
	anterior chamber cells, n (%)	7 (38.89%)	4(30.77%)	8 (34.78%)	3 (50.00%)	22 (36.67%)
	anterior chamber flare, n (%)	4 (22.22%)	3 (23.08%)	3 (13.04%)	2 (33.33%)	12 (20.00%)
Posterior segment	Vitritis, n (%)	2 (11.11%)	4(30.77%)	7 (30.43%)	3 (50.00%)	18 (30.00%)
	Choroidal effusion, n (%)			2 (8.70%)		2 (3.33%)
	Chorioretinopathy, n (%)	1 (5.56%)				1 (1.67%)
	macular edema, n (%)	3 (16.67%)	2 (15.38%)	4 (17.39%)		9 (15.00%)
	Papillitis, n (%)	1 (5.56%)	4 (30.77%)	6 (26.09%)		11(18.33%)
	subretinal fluid, n (%)		1 (7.69%)	2 (8.70%)	1 (16.67%)	4 (6.67%)
	Serous retinal detachment, n (%)	1 (5.56%)	2 (15.38%)	2 (8.70%)		5 (8.33%)

% Algorithm: The number of cases with positive signs divided by the total number of cases using the drug; SD: Standard Deviation; VKH: Vogt-Koyanagi-Harada Syndrome.

**Table 3:** Clinical manifestations of uveitis caused by specific targeted therapy.

Clinical Manifestations		Erlotinib	Trametinib and Dabrafenib	Vemurafenib	Nilotinib	MEK Inhibitor XL518	Encorafenb and Binimetinib	Total
Total, n		3	6	12	1	3	1	26
Sex	Male, n (%)		4 (66.67%)	6 (50.00%)	1 (100%)	1 (33.33%)		12 (46.15%)
	Female, n (%)	3 (100%)	2 (33.33%)	6 (50.00%)		2 (66.67%)	1 (100%)	14(53.85%)
Mean Age, years ± SD		69 ± 5.89	53 ± 12.91	55.17 ± 13.95	77	56.67 ± 4.71	81	58.27 ± 13.88
Bilateral involvement, n (%)		3 (100%)	5 (83.33%)	9 (75.00%)	1 (100%)	3 (100%)	1 (100%)	22 (84.62%)
Simultaneous onset of both eyes, n (%)		3 (100%)	5 (83.33%)	9 (75.00%)	1 (100%)	3 (100%)	1 (100%)	22(84.62%)
Mean time from the initiation of treatment to onset of uveitis, weeks ± SD		6	8.25 ± 5.79	39.91 ± 38.64	24	2 ± 1.41	24	21.62 ± 22.73
First symptoms	Vision loss, n (%)	1 (33.33%)	4 (66.67%)	6 (50.00%)	1 (100%)	1 (33.33%)	1 (100%)	14 (53.85%)
	Red and pain, n (%)	1 (33.33%)		4 (33.33%)				5 (19.23%)
	Floaters, n (%)	1 (33.33%)		1 (8.33%)				2 (7.69%)
	Visual field defect, n (%)							
systemic condition	VKH-like systemic manifestations, n (%)			1 (8.33%)			1 (100%)	2 (7.69%)
	Hypophysitis, n (%)							
	colitis, n (%)							
	Autoimmune hepatitis, n (%)							
	pain, n (%)	1 (33.33%)						1(3.85%)
Anterior segment	posterior synechiae, n (%)		2 (33.33%)	4 (33.33%)				6 (23.08%)
	keratic precipitates, n (%)	1 (33.33%)	2 (33.33%)	1 (8.33%)			1 (100%)	5 (19.23%)
	fibrin formation, n (%)							
	anterior chamber cells, n (%)	1 (33.33%)	2 (33.33%)	5 (41.67%)				8 (30.77%)
	anterior chamber flare, n (%)	1 (33.33%)						1 (3.85%)
Posterior segment	Vitritis, n (%)	1 (33.33%)	2 (33.33%)	3 (25.00%)				6 (23.08%)
	Choroidal effusion, n (%)							
	Chorioretinopathy, n (%)							
	macular edema, n (%)			4 (33.33%)				4 (15.38%)
	Papillitis, n (%)		1 (16.67%)	1 (8.33%)				2 (7.69%)
	subretinal fluid, n (%)		2 (33.33%)	1 (8.33%)				3 (11.54%)
	Serous retinal detachment, n (%)		1 (16.79%)	2 (16.67%)		2 (66.66%)	1 (100%)	6 (23.08%)

% algorithm: The number of cases with positive signs divided by the total number of cases using the drug.

**Table 4:** Comparison of clinical features of uveitis induced by immunotherapy and specific targeted therapy.

Characteristics		Immunotherapy (*n* = 60)	Percent (%)	Specific Targeted Therapy (*n* = 26)	Percent (%)	*p*-Value		
						Chi-Square Test	Fisher Exact Test	Independent-Samples T Test
Sex	Male Female No/Unknown	28 30 2	46.7 50.0 3.3	12 14 0	46.2 53.9 0.0	0.857	0.523	
Age	<60 years ≥60 years	28 32	46.7 53.3	12 14	46.2 53.9	0.965	0.577	
Bilateral involvement	Yes No	48 12	80.0 20.0	22 4	84.6 15.4	0.613	0.430	
Average onset time (weeks ± SD)		13.8 ± 19.4		21.6 ± 22.7				0.165
Average visual acuity at visit (n ± SD)		0.3 ± 0.5		0.7 ± 1.4				0.107
Type of uveitis	Anterior	24	40.0	8	30.8	0.416	0.286	
	Intermediate	2	3.3	8	30.8	0.001	<0.001	
	Posterior	8	13.3	3	11.5	0.819	1.000	
	Panuveitis	17	28.3	4	15.4	0.199	0.156	
First symptoms	Vision loss	17	28.3	15	57.7	0.010	0.010	
	Red and pain	8	13.3	6	23.1	0.420	0.207	
	Floaters	5	8.3	3	11.5	0.948	0.456	
	Visual field defect	1	1.7	0	0.0	0.508	0.698	
Systemic condition	VKH-like systemic manifestations	4	6.7	2	7.7	0.864	0.591	
	Hypophysitis	3	5.0	0	0.0	0.246	0.550	
	Colitis	2	3.3	0	0.0	0.346	0.484	
	Autoimmune hepatitis	1	1.7	0	0.0	0.508	0.698	
	Pain	0	0.0	1	3.9	0.127	0.302	
Anterior segment	Posterior synechiae	7	11.7	6	23.1	0.304	0.152	
	Keratic precipitates	5	8.3	5	19.2	0.279	0.140	
	Fibrin formation	2	3.3	0	0.0	0.346	0.484	
	Anterior chamber cells	22	36.7	8	30.8	0.598	0.393	
	Anterior chamber flare	12	20.0	1	3.9	0.111	0.097	
Posterior segment	Vitritis	18	30.0	6	23.1	0.511	0.352	
	Choroidal effusion	2	3.3	0	0.0	0.346	0.484	
	Chorioretinopathy	1	1.7	0	0.0	0.508	0.698	
	Macular edema	9	15.0	4	15.4	0.964	1.000	
	Papillitis	11	18.3	2	7.7	0.349	0.176	
	Subretinal fluid	4	6.7	3	11.5	0.742	0.356	
	Serous retinal detachment	5	8.3	6	23.1	0.126	0.067	

## Data Availability

Not applicable.
